# Ketogenic Diets and Hepatocellular Carcinoma

**DOI:** 10.3389/fonc.2022.879205

**Published:** 2022-05-04

**Authors:** Yan Lan, Chaonan Jin, Pavitra Kumar, Xia Yu, Cameron Lenahan, Jifang Sheng

**Affiliations:** ^1^ State Key Laboratory for Diagnosis and Treatment of Infectious Diseases, National Clinical Research Center for Infectious Diseases, Collaborative Innovation Center for Diagnosis and Treatment of Infectious Diseases, The First Affiliated Hospital, College of Medicine, Zhejiang University, Hangzhou, China; ^2^ Department for BioMedical Research, Hepatology, University of Bern, Bern, Switzerland; ^3^ Department of Biomedical Science, Burrell College of Osteopathic Medicine, Las Cruces, NM, United States

**Keywords:** ketogenic body, ketogenic diet, cancer cell metabolism, hepatocellular carcinoma, liver

## Abstract

The ketogenic diet (KD) is a low-carbohydrate, high-fat diet regarded as a potential intervention for cancers owing to its effects on tumor metabolism and behavior. Hepatocellular carcinoma (HCC) is the most prevalent type of liver cancer, and its management is worth investigating because of the high fatality rate. Additionally, as the liver is the glucose and lipid metabolism center where ketone bodies are produced, the application of KD to combat HCC is promising. Prior studies have reported that KD could reduce the energy supply and affect the proliferation and differentiation of cancer cells by lowering the blood glucose and insulin levels. Furthermore, KD can increase the expression of hydroxymethylglutaryl-CoA synthase 2 (HMGCS2) in hepatocytes and regulate lipid metabolism to inhibit the progression of HCC. In addition, β-hydroxybutyrate can induce histone hyperacetylation and reduce the expression of inflammatory factors to alleviate damage to hepatocytes. However, there are few relevant studies at present, and the specific effects and safety of KD on HCC warrant further research. Optimizing the composition of KD and combining it with other therapies to enhance its anti-cancer effects warrant further exploration.

## Introduction

Liver cancer deaths account for the third-highest number of cancer-related fatalities worldwide and rank sixth in the number of new cancer cases, thereby exerting a substantial impact on human health and the economy (1). According to World Health Organization estimates, over one million individuals will die from liver cancer in 2030 (2). Hepatocellular carcinoma (HCC) accounts for 75%–85% of all liver cancer cases, and the risk factors vary from one region to another. In Asia, hepatitis B virus (HBV) and aflatoxin exposure are the major causes, whereas the hepatitis C virus is the leading cause in Europe. In addition, because of the differences in diet and lifestyle, non-alcoholic fatty liver disease (NAFLD) accounts for 10%–20% of the HCC cases in the United States (3). In past decades, therapies for HCC have witnessed rapid progress, with the main options being surgical resection, orthotopic liver transplantation, radio-frequency ablation, chemotherapy, radiotherapy, and systemic drugs such as sorafenib (2). However, the prognosis of patients with HCC remains poor, and more efficient treatments with fewer side effects have yet to be proposed (4). Cancer cells have different metabolic characteristics when compared with normal cells. While the normal cells obtain energy through glycolysis and the citric acid cycle (TCA/Krebs cycle), cancer cells undergo increased glycolysis and use most of the pyruvate generated through this process for lactate production even when oxygen is available (5). The Warburg effect is a characteristic metabolism in tumor cells, in which cells shift the source of ATP production from oxidative phosphorylation to rapid anaerobic glycolysis. Therefore, Warburg effect promotes glucose uptake, reduces the amount of ATP produced, but increases the rate of ATP production and substances required for cell growth, and prevents apoptosis and immune escape (6). The Warburg effect has encouraged many scientists to determine whether tumor growth can interfere with metabolic pathways. Accordingly, the ketogenic diet (KD), a low-carbohydrate, high-fat diet proposed in 1920 to treat intractable epilepsy was found to be capable of reducing glucose metabolism and increasing lipid metabolism, thereby interfering with the Warburg effect and preventing tumor cell growth. This diet was later proven to have promising anti-cancer effects (7).

The anti-cancer effect of KD has been demonstrated in several clinical studies conducted in many types of tumors, such as glioblastoma, advanced malignant astrocytoma, invasive rectal cancer, breast cancer, ovarian cancer, and endometrial cancer. Additionally, KD application has led to improvements in metabolic indicators, such as total cholesterol, low-density lipoprotein (LDL), and insulin levels in patients with cancer (8). KD has alleviated cachexia in patients and improved their quality of life (9, 10). Notably, a clinical study has reported adverse effects of hyperuricemia, but this effect was associated with low patient compliance (11). Most clinical studies have not documented any serious adverse events linked to KD. However, the effect of KD on tumors is also influenced by the tumor type (12). For example, unlike the anti-cancer effects observed in prior studies, a high-fat ketogenic diet was found to promote tumor growth in a mouse model of melanoma (13). As the main metabolic organ in the body, the liver plays a crucial role in the metabolism of carbohydrates, lipids, and proteins. Furthermore, the liver acts as the site of ketone body (KB) production, and abnormalities in liver function in patients with HCC can also have an impact on metabolism (14). Hydroxymethylglutaryl-CoA synthase 2 (HMGCS2) is the key enzyme in the ketogenic process, but is not expressed in fetal liver. However, it is activated in adult liver. Studies have found that low HMGCS2 expression is associated with advanced clinical stage and poor prognosis in HCC (15). Therefore, it is especially necessary to understand the potential therapeutic role of KD in HCC treatment. A study by Healy et al. noted that KD reduced the tumor load in HCC mice (16). However, according to Byrne’s research, KD had no significant effect on the progression of HCC in mice (17). In conclusion, the specific effects of KD on HCC are unclear. Hence, in this review, we have summarized the metabolism of KBs, the types of ketogenic diets, the possible mechanisms of action of KD on HCC, and the data obtained from current pre-clinical and clinical studies, with a hope of providing a strong theoretical basis for subsequent studies.

## Anabolic and Catabolic Metabolism of Ketone Bodies

Liver mitochondria are the site of KB production; KB mainly includes acetoacetate (AcAc), β-hydroxybutyrate (β-OHB), and acetone (Ac). Under normal conditions, the blood ketone levels range from 0.1–0.2 mM. KB production increases during starvation, long-term exercise, or diabetes, and blood ketones can reach a level as high as 1 mM after 24 hours of fasting (18). First, fatty acids (FAs) are catabolized into acetyl-CoA through β-oxidation, which then enter the citric acid cycle and release energy. Nonetheless, in the low-glucose state, acetyl-CoA is used for KB synthesis. Two molecules of acetyl-CoA are condensed to form acetoacetyl-CoA (AcAc-CoA) by acetoacetyl-CoA thiolase (ACAT1) and AcAc-CoA is used in the generation of β-hydroxy-β-methylglutaryl-CoA (HMG-CoA) by HMG-CoA synthase (HMGCS2), the rate-limiting enzyme in ketogenesis. Subsequently, HMG-CoA lyase degrades HMG-CoA into AcAc, which is further reduced to β-OHB under the action of β-hydroxybutyrate dehydrogenase (BDH). The above two KBs are the main forms used by the body. A fraction of the AcAc spontaneously undergoes decarboxylation to form acetone, which is excreted through urine or respiration, or is further metabolized into pyruvate, lactic acid, and acetic acid (19).

**Figure 1:**
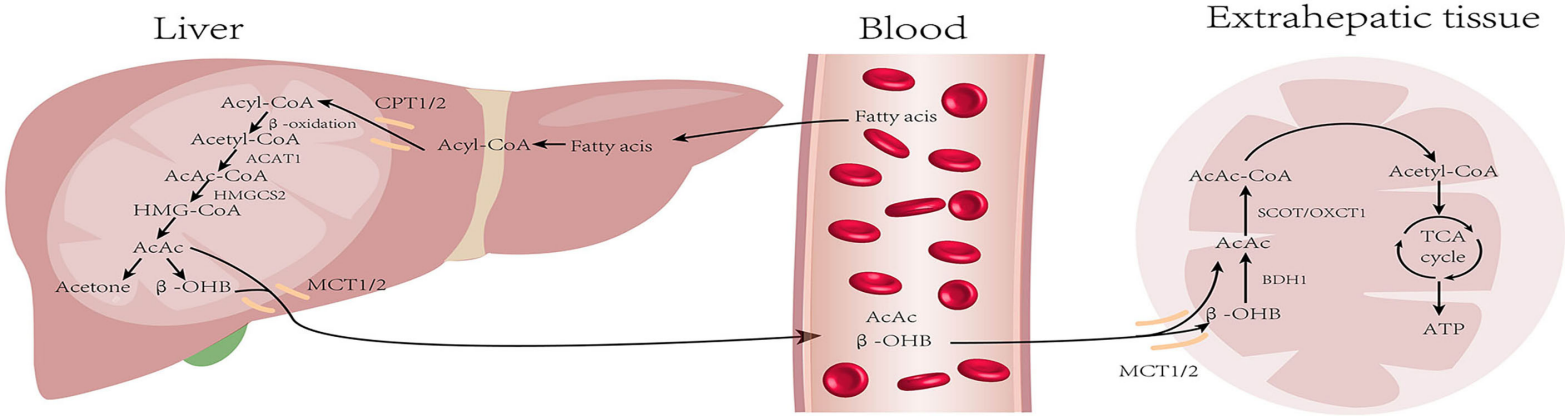
Metabolism of ketone bodies: During starvation and prolonged exercise, lipid hydrolysis is enhanced and fatty acid production increases. First, fatty acids (FAs) are activated into acyl-CoA and then decomposed into acetyl-CoA through β-oxidation. Acetyl-CoA is converted into ketone bodies in mitochondria within hepatocytes. Then, ketone bodies are transported by blood and utilized by extrahepatic tissues (heart, brain, kidney, and skeletal muscle) to regenerate acetyl-CoA. Finally, acetyl-CoA is oxidized through the tricarboxylic acid cycle for energy supply. (CPT, carnitin-palmitoyl transferase; MCT, monocarboxylate transporter).

The liver can not oxidize KBs. Therefore, the KBs produced in the liver are transported through the bloodstream to be oxidized and utilized in extrahepatic tissues such as the heart, kidney, brain, and skeletal muscle. The β-OHB in the bloodstream is taken up by the mitochondria through the monocarboxylate transporter protein (MCT), and is then dehydrogenated to produce AcAc in the presence of BDH1. The key enzyme 3-oxoacid transferase 1 (OXCT1), also called succinyl CoA transferase (SCOT), converts AcAc and succinyl CoA to AcAc-CoA (20). AcAc-CoA is then converted to two molecules of acetyl CoA by the action of ACAT1. Finally, acetyl CoA is either oxidized and utilized *via* the TCA cycle or it is used for cholesterol synthesis (21) **(** **Figure 1** **)**.

## Ketogenic Diet

The classical KD consists of a 4:1 or 3:1 ratio of fat content to carbohydrate plus protein, and requires all ingredients to be calculated and weighed precisely in grams (22). The fatty acids in the classical KD are long-chain triglycerides (LCT), which must be converted in the body before absorption. Later, Huttenlocher et al. proposed the medium-chain triglyceride ketogenic diet (MKD), which is more easily digested and absorbed than LCT, as medium-chain triglycerides (MCT) do not require emulsification, and can be directly degraded into glycerol and water-soluble medium-chain fatty acids (MCFA) (23). However, because the main component of the classical KD and MKD is fat, patients often experience some gastrointestinal disturbances, such as diarrhea and vomiting; hence, compliance is poor (24). Therefore, researchers have recommended a modified Atkins diet (MAD) and low glycemic index therapy (LGIT) ketogenic diet based on the classical KD (25–31) **(** **Table 1** **)**.

**Table 1:** Types, characteristics and clinical research of ketogenic diet.

Types	The classical ketogenic diet	MKD	MAD	LGIT Ketogenic Diet
**Components**	Mainly long-chain triglycerides, 90% of calories come from fat, 8% protein, and only 2% carbohydrate.	Replace long-chain triglycerides with medium-chain triglycerides.	Fat: carbohydrates and proteins are close to 0.9:1, and about 65% of calories come from fat.	Carbohydrate intake is limited to foods with less blood glucose, such as non-starchy vegetables.
**Dietary requirements**	Strict.	Strict.	Stricter requirements for carbohydrate intake, no restrictions on fat and protein.	Total carbohydrate intake is less strict.
**Patient compliance**	Poor.	Poor.	Better.	Better.
**Clinical research and the time for KD therapy**	Pancreas, Lung: 2 days before chemotherapy and radiation, last for 5.5 – 7 weeks (11).	Breast: Started at the same time as chemotherapy, last for 90 days (28, 29); HNC: Started at the same time as chemotherapy, last for 39 days; Rectal: Started concurrently with radiotherapy, last for 39 days (30).	Glioma: Started at the same time as chemotherapy and radiotherapy, last for 6 week (31).	

KD, Ketogenic diet; HNC, Head and neck cancer; LGIT, Low glycemic index therapeutic; MAD, Modified atkins diet; MKD, Medium chain triglyceride ketogenic diet.

## Potential Effects of KD on Hepatocellular Carcinoma

### Regulation of Glucose Metabolism

**Figure 2:**
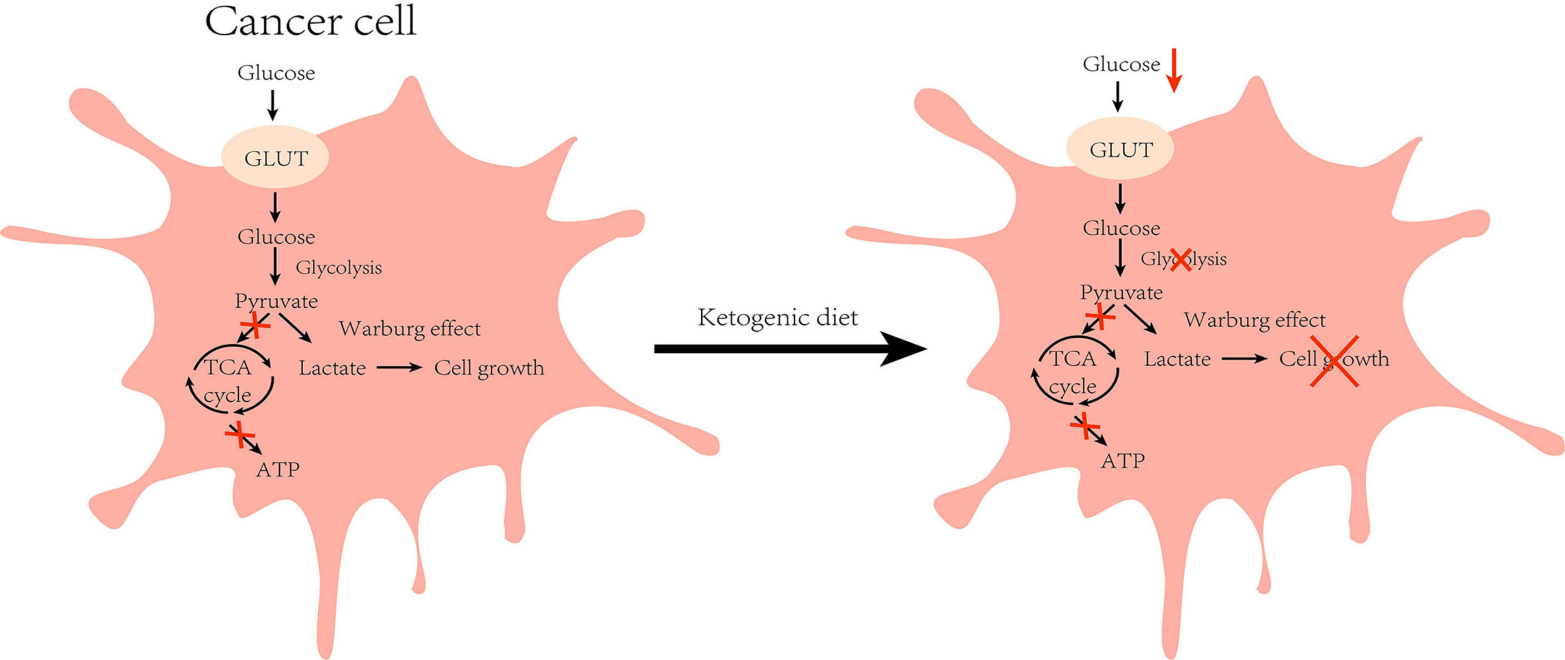
Effect of ketogenic diet on glucose metabolism in cancer cell: Cancer cells are characterized by higher glycolysis due to defective mitochondrial function, and the accumulated cytosolic pyruvate is used to produce lactate (Warburg effect) during normal diet. However, under ketogenic diet feeding, glucose intake is reduced and the growth of cancer cells is inhibited by reduced energy supply. (GLUT, glucose transporter).

Tumor cells are characterized by rapid growth and high metabolism, and glucose transport protein (GLUT) aids in the entry of glucose into the cells for metabolism to enable cell growth. It has been found that GLUT1 expression is enhanced in HCC and exerts tumorigenic effects (32). Experiments on HCC mice revealed that the tumor incidence was 3–5 times higher in mice fed with a high-glucose diet when compared with those in the normal diet group (33). These findings indicate that glucose metabolism is significant for the development of HCC. In the absence of glucose availability, normal cells synthesize KBs in the mitochondria to supply energy for maintaining cell growth. However, tumor cells cannot perform ketone metabolism because of abnormalities in mitochondrial structure and function (34) **(** **Figure 2** **)**. Moreover, the activities of SCOT and ACAT1, key enzymes in ketone metabolism, were low or undetectable in the tumor tissues, which confirmed the above idea (35). KD is a low-carbohydrate diet that reduces the energy supply to the cancer cells by lowering blood glucose levels in the body to exert anti-cancer effects. Clinical trials have demonstrated that insulin and insulin-like growth factor-1 (IGF-1) levels decreased with the consumption of KD for a period of time, and were negatively correlated with serum β-hydroxybutyric acid levels (36, 37). Indeed, insulin and IGF-1 play crucial regulatory roles in human growth and metabolism, and epidemiological evidence demonstrates that they can promote tumorigenesis and progression by augmenting tumor cell proliferation, migration, and angiogenesis (38). In addition, an HCC mouse trial showed a positive correlation between tumor load and post-prandial serum insulin levels (16). The phosphoinositide 3-kinase (PI3K) pathway is an intracellular signal transduction pathway that promotes cell proliferation and differentiation. Several inhibitors targeting this pathway are presently being investigated for the inhibition of tumor growth. However, clinical data allude that drugs targeting PI3K can cause adverse effects, such as hyperglycemia, thereby reactivating the PI3K pathway and diminishing the therapeutic effect (39). To address this issue, Hopkins et al. performed experiments on mouse tumor models. They found that the use of PI3K inhibitors in conjunction with a ketogenic diet inhibited the insulin feedback-induced activation of the mTORC1 pathway by lowering insulin levels, thereby enhancing the efficacy of PI3K inhibitors, resulting in an anti-tumor effect (40). The above-mentioned results suggest that KD can exert anti-cancer effects by regulating the process of glucose metabolism to reduce the energy supply of cells, thus inhibiting the growth of the cancer cells.

### Regulation of Lipid Metabolism

The liver is the site of lipid metabolism, and lipid metabolism tends to affect liver function. Abnormalities in lipid metabolism can cause NAFLD, which in turn can lead to the development of HCC. Under a normal diet, acetyl-CoA produced by the TCA cycle is converted into malonyl-CoA by ACC (the key enzyme in fatty acid synthesis), and then finally converted into fatty acids through a series of biosynthetic processes. Similarly, cholesterol biosynthesis also begins with acetyl-CoA, and the rate-limiting enzyme for its synthesis is HMGCS2 reductase (HMGCR). However under the KD, insufficient energy metabolism and low insulin inhibit the activity of ACC and HMGCR, leading to reduced lipid synthesis (41). Restriction of carbohydrate intake is adopted as a dietary intervention for patients with obesity, and studies have found multiple benefits with regard to NAFLD (42). Numerous clinical studies have established the positive effects of KD on patients with obesity and NAFLD. In a 6-day intervention involving 10 patients with obesity, Luukkonen et al. found that KD promoted hydrolysis of intrahepatic triglycerides (IHTG), improved insulin resistance, and decreased the serum insulin level in patients, resulting in decreased IHTG and body weight (43). In addition, Browning et al. performed a 2-week dietary intervention in patients with NAFLD and noted that the low-carbohydrate diet was more beneficial than the low-calorie diet in reducing hepatic triglyceride (TG) levels (44). Yancy et al. conducted a dietary intervention on 120 overweight, hyperlipidemic volunteers for 6 months, and observed that when compared with the low-fat diet, the low-carbohydrate diet was not only more effective in reducing the serum TG levels, but also in significantly lowering body weight and increasing the high-density lipoprotein cholesterol (HDL-C) levels (45). Foster et al. conducted long-term intervention in patients with obesity. While the results of their short-term trials matched the findings of Yancy et al., their results at 2 years found that only HDL-C levels were higher than those in the low-fat diet group, with no apparent variations in any other indicators (46). These results suggest that the effect of low carbohydrates on patients is influenced by the duration of the diet, and that prolonged intervention studies are needed to further illustrate the follow-up impact. Of note, not all low-carbohydrate diets are KD (47). The low-carbohydrate group in the above studies strictly controlled their carbohydrate intake, all of which were <20g/day. However, there were no strict restrictions regarding fat and protein contents, which met the requirements of MAD. It is worth noting that after feeding mice on a very low-carbohydrate ketogenic diet for 12 weeks, Garbow et al. found that the mice developed hepatic endoplasmic reticulum stress, steatosis, and cellular damage, causing a pattern of damage similar to the NAFLD phenotype, however, the occurrence of this phenomenon might have been the result of extreme KD (95.1% of calories from fat, 0.4% from carbohydrate, and 4.5% from protein) (48). In addition to this, some studies show that KD leads to systemic glucose intolerance and the development of NAFLD (49, 50). One case reported a woman who developed potential NAFLD after the KD with elevated liver enzymes (51). From these findings, it could be inferred that KD with various composition ratios can produce diverse or even opposing results. Watanabe et al. analyzed the current articles on KD and NAFLD, and found that a high-fat ketogenic diet (HFKD) significantly affects liver fat reduction. Nevertheless, this effect was evident in the short- and medium-term only, but diminished with time. Additionally, very low-calorie KD (VLCKD) can reduce body weight and improve hepatic steatosis (47). The above studies illustrate that the regulation of lipid metabolism by KD is influenced by several factors. At present, there are few articles about the KD as a risk factor for NAFLD. In general, KD exerts a positive effect on NAFLD. Since KD can regulate lipid metabolism and reduce body weight, it has shown a positive effect on cardiovascular function, and studies have also found that KD has a certain improvement in systolic and diastolic blood pressure (52, 53).

**Figure 3:**
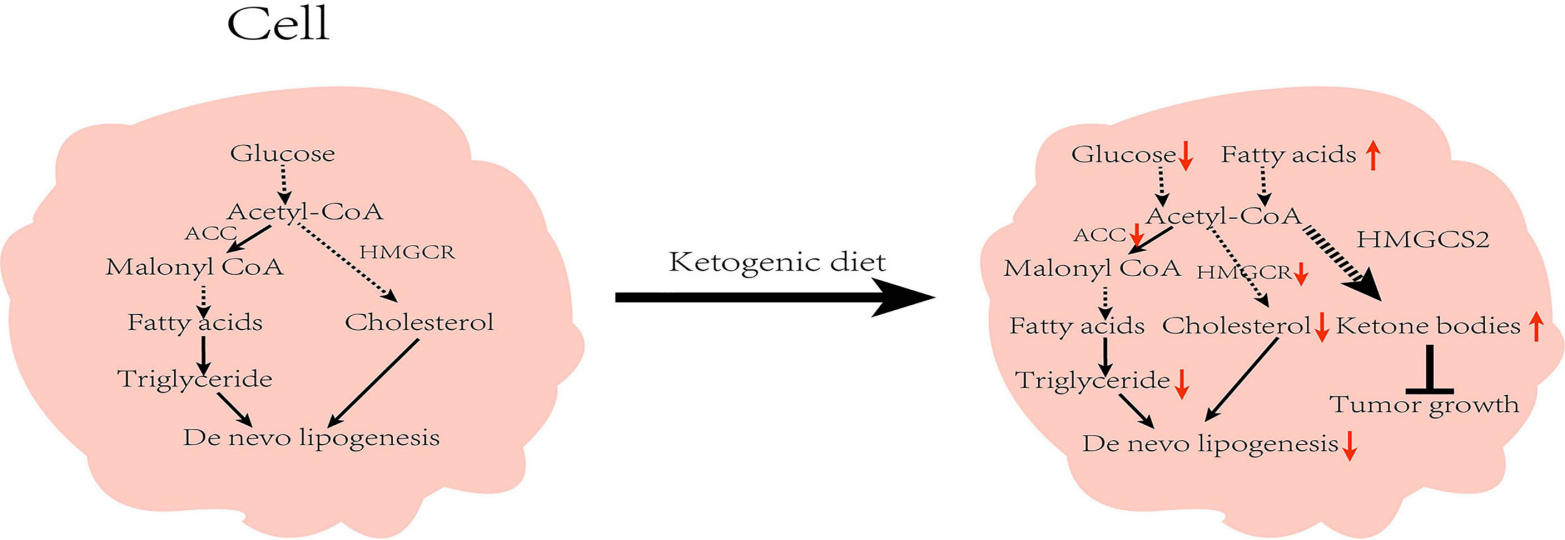
Effect of ketogenic diet on lipid metabolism in cells: While on the ketogenic diet, carbohydrates intake is reduced and the activity of acetyl-CoA carboxylase and β-hydroxy-β-methylglutaryl-CoA reductase are inhibited, which in turn reduces *de novo* lipid synthesis. Concurrently, increased fat mobilization and ketone body production inhibit tumor growth. ACC, Acetyl-CoA carboxylase; HMGCR, β-hydroxy-β-methylglutaryl-CoA reductase; HMGCS2; HMG-CoA synthase.

Moreover, many key enzyme inhibitors in lipid metabolism have demonstrated anti-cancer effects (54). HMGCS2 is an important enzyme in fatty acid oxidation (55). A pre-clinical study has demonstrated that HMGCS2 deficiency causes ketogenic disorders, leading to extensive hepatocellular damage and inflammation. Furthermore, the deficiency has been shown to result in hepatic TCA circulatory disturbances and promote the development of NAFLD (56). Through immunohistochemical staining, Wang et al. noted that HMGCS2 was highly expressed in healthy liver tissues. However, the expression was significantly decreased in liver cirrhosis and in HCC tissues. Also, the expression of HMGCS2 was negatively associated with the pathological grade and clinical stage of HCC. Knock-down of HMGCS2 promoted cell proliferation by enhancing the c-Myc/cyclinD1 pathway and inhibiting the caspase-dependent apoptosis pathway. The knockdown also up-regulated the epithelial–mesenchymal transition signal to enhance cell migration and promote tumorigenesis. Interestingly, supplementation of β-OHB to cells in which HMGCS2 was knocked down revealed a decrease in proliferation and migration of HCC cells. Mice fed with KD also had lower subcutaneous tumor growth rates than those on a standard diet (57). Furthermore, Wang et al. investigated the xenograft mouse mode (58). They were surprised to find that the tumors of KD-fed mice expressed higher levels of the HMGCS2 protein than those on a normal diet, and that the tumor size in the KD-fed mice group was inversely associated with HMGCS2 protein expression. These results demonstrate that KD can exert anti-HCC effects by enhancing the expression of HMGCS2 **(** **Figure 3** **)**.

### Regulation of Inflammatory Response

When compared with normal cells, more reactive oxygen species (ROS) are produced in tumor cells because of defective mitochondrial oxidative phosphorylation, which causes cellular oxidative damage (59). Studies have indicated that inflammation has a promotional effect on tumorigenesis and development, and many antagonists targeting inflammatory cytokines are being developed and investigated to prevent and treat cancer (60, 61). Viral hepatitis is a major cause of HCC, and it has been confirmed that inflammation can promote HCC development in multiple ways (62). Therefore, suppressing the inflammatory response of hepatocytes is an essential mechanism to combat HCC. β-OHB, the main component of KBs, has a butyrate-like structure, and is an endogenous inhibitor of histone deacetylase. The effect of β-OHB is dose-dependent. KD can augment serum β-OHB levels and exert anti-inflammatory and anti-tumor effects by inducing histone hyperacetylation (35). The anti-inflammatory effects of KBs have also been confirmed by analyzing mouse microglia. β-OHB may exert its anti-inflammatory effects by activating GPR109A and by inhibiting the NF-κB pathway, which decreases the expression of inflammatory factors, such as COX-2 (63). GPR109A is a protein receptor that inhibits tumor growth; β-OHB is a physiological agonist of GPR109A that exerts its tumor-suppressive effects by agonizing GPR109A (64). Moreover, a study found that the ketogenic diet can also produce anti-inflammatory effects by inhibiting the assembly of the NLRP3 inflammasome, a complex that can control the release of cytokines, such as tumor necrosis factor-alpha (TNF-α). Besides, TNF-α can interfere with insulin signaling, leading to insulin resistance (65). Many reports have shown that β-OHB exerts significant anti-inflammatory effects. By evaluating the effects of different β-OHB levels on cow hepatocytes, it was found that β-OHB induces activation of the NF-κB signaling pathway by up-regulating oxidative markers and down-regulating antioxidant markers, thereby causing inflammatory damage to the cells and increasing the oxidative status index with increasing β-OHB levels (66). Therefore, we speculate that the contradiction of the above results may be related to the level of β-OHB and cell type, as well as other factors. AcAc, another KB, was proven to promote oxidative stress in cellular assays, leading to cellular damage through the up-regulation of NADPH oxidase 4 (NOX4) expression and NADPH oxidase activity. The effect of ROS was stronger at 25 mM glucose than with KB alone (67). In response to some controversies about ROS production by KB, Meroni et al. separately examined cells with β-OHB and AcAc, and observed that KB activates the transcription factor, Nrf2, by inducing moderate oxidative stress, which further activates antioxidant defense systems to prevent damage (68). Collectively, the specific effect of KB on the inflammatory response remains unclear, but the types of KB and cell appear to be factors that influence its anti-inflammatory effect. More trials are necessary to pursue a greater in-depth understanding.

## Pre-Clinical and Clinical Trials

Several pre-clinical trials have established KD as an effective anti-cancer strategy to delay tumor growth and prolong survival time, but the effect of KD on tumors is influenced by tumor type (69, 70). Little research has been conducted regarding KD and HCC. Huang et al. observed that serum glucose levels and body weight were significantly reduced in mice fed with KD. However, HCC cells under starvation can trigger the mTORC2-AKT-SP1 signaling, which promotes OXCT1 (a key ketolytic enzyme) expression and inhibits AMPK-mediated autophagy, thus protecting the cancer cells. The elevated KBs *in vivo* can promote HCC cell growth (71). OXCT1 is not routinely expressed in the adult liver (20). The above results may indicate a metabolic adaptation of HCC cells in the presence of nutritional deficiency. Most importantly, the above study reveals a positive relationship between OXCT1 expression and the clinical stage of HCC. Patients with high OXCT1 expression have a short survival time, which signifies that OXCT1 holds promise as a new biomarker for HCC. Moreover, studies have shown that the effect of KD on HCC mice is also affected by the intervention time. Diet interventions were started in the HCC mouse model in the 6th week, and the tumor burden and diversity of the mice in the KD-fed group were lower than those in the high-glucose diet group (16). However, when dietary intervention commenced at 40 weeks, KD did not have a significant effect on HCC (17), suggesting that early intervention with KD is beneficial in HCC. The above result is consistent with findings of previous studies. A meta-analysis showed that the timing of dietary intervention affects the therapeutic effect of KD, and that prophylactic KD intervention has a strong anti-cancer effect (72).

Clinical studies related to the impacts of KD on HCC are limited, and there are only two case reports on liver tumors (73). After applying KD monotherapy for 1 month, a patient’s blood glucose level decreased to normal, and ultrasound showed that the tumor had disappeared after 1 year of KD. The other patient also demonstrated a significant improvement in clinical performance. Furthermore, no serious side effects were reported during the therapeutic period. However, the specifics of this case report, such as the stage of the patient’s tumor and the specific composition of the KD, were not described, all of which can significantly impact treatment outcome. In addition, the small number of participants is a shortcoming of the report. Hence, the application of KD for treating HCC still lacks proper evidence to support it, and more clinical studies are needed to provide confirmation.

## Adverse Effects of KD in Treating Cancer

Although most studies have proved the safety of KD in the treatment of cancer, some adverse effects related to KD have been reported in the literature, namely, gastrointestinal reactions, increased blood lipid levels, kidney stones, and fractures (12, 74). For example, Mansoor et al. found that a low-carbohydrate dietary intervention for more than 6 months in healthy people increased their LDL-C levels (75). Similarly, a 60-day KD intervention in rats resulted in several adverse effects, such as anemia, metabolic acidosis, and decreased plasma superoxide dismutase (SOD) levels (76). In addition, studies exploring carbon tetrachloride- and thioacetamide-induced liver fibrosis mouse models revealed that KD leads to the development of cirrhosis by increasing cholesterol content in the liver, which subsequently enhances the hepatic inflammatory response and decreases the antioxidant and detoxification capacity (77) (50).Most of the above-mentioned adverse events are important factors affecting HCC, which promote its occurrence through glucose metabolism, inflammatory response, and increased NAFLD mechanisms. Moreover, the ketogenic diet is different from the traditional diet in that the patient needs to prepare the meal correctly and cannot eat with friends and family normally, which can make the patient feel socially isolated. In addition, the cost of the patient’s diet due to the change in food composition will also change. These can contribute to psychosocial problems for the patient. Therefore, we should pay attention to these adverse reactions in future studies to ensure the safety and feasibility of KD.

## Potential Future Research Directions

There are increasing reports of combination therapies against progression of various tumors, including HCC (78, 79). Similarly, the ketogenic diet could be combined with a secondary drug to enhance its therapeutic potential against HCC. For example, inhibitors of the mevalonate pathway could control the elevated cholesterol in the blood and liver after KD. It is well established that the rate-limiting step in cholesterol synthesis is the conversion of hydroxyl-methyl glutaryl-coenzyme A (HMG-CoA) into mevalonate. In fact, small molecule inhibitors of the mevalonate pathway increased the cell death of liver carcinoma cells in several *in vitro* studies (80–82). Statins are a classic inhibitor of mevalonate pathway, which are clinically used to reduce hypercholesterolemia. Several studies have found that statins can significantly reduce the risk of HCC (83). Furthermore, Thrift et al. found that statins also reduced mortality in HCC patients (84). Therefore, we suggest the use of KD combined with the inhibitors of mevalonate pathway such as statins for preclinical and clinical studies.

Recently, KD has been shown to be beneficial in treating many diseases, but different KD compositions tend to show varying effects. For example, in a 45-day randomized trial, significant improvements in weight, blood pressure, and some metabolic parameters were seen in three groups on a VLCKD containing whey, vegetable, or animal proteins. However, whey and vegetable proteins had a better safety profile than animal proteins, and whey protein demonstrated the strongest effect in regulating the gut microbiota and maintaining muscle performance (85). In addition, some large prospective cohort studies have confirmed the superiority of plant proteins, revealing that plant protein intake is negatively related to all-cause mortality. The replacement of animal proteins, especially processed and red meat proteins, with plant proteins reduces cancer and cardiovascular disease-related mortality (86, 87). These results could be attributed to the fact that plant proteins can improve insulin sensitivity and lower blood pressure and LDL levels. In the two pre-clinical trials on HCC discussed above, the proteins included in the KD were animal proteins. Therefore, the type of protein was not an influencing factor in the different effects (16, 17). However, one cannot help but wonder if KD containing plant proteins would yield more surprising results if it was used to intervene in HCC mice. In addition to the type of protein, the role played by the type of fat ingested can vary. Both the classical KD and MKD showed inhibitory effects on tumor growth when compared with the standard diet. However, the MKD containing omega-3 and medium-chain triglycerides had a more pronounced effect than the classical KD (88). The findings from these pre-clinical studies suggest that diet optimization should be performed in future studies to achieve the optimal anti-cancer effect of KD.

Owing to the abnormal mitochondrial function, the production of ROS is increased in tumor cells during mitochondrial respiration. Simultaneously, KD limits the regeneration of NADPH by converting sugar metabolism to lipid metabolism and by further increasing the oxidative stress level of the tumor cells (89). Because cancer cells have a higher oxidative stress response than the normal cells, as well as a higher sensitivity to radiotherapy and chemotherapy, KD can be used as an adjuvant therapy to kill the tumor cells selectively. It has been demonstrated that KD can enhance the effects of chemotherapy and radiotherapy in brain tumors (70). In addition, a prospective clinical trial involving nine patients supported the idea that KD is safe and feasible as an adjuvant in the standard treatment for glioblastoma (90). Similarly, women with breast cancer who received radiotherapy and KD had better metabolic indicators and a significant improvement in quality of life when compared with those on a standard diet (91). Radiotherapy is a critical treatment for HCC. Therefore, combining it with KD to improve treatment outcome holds promise.

## Conclusion

Currently, treatment options for HCC are limited, and better therapeutic options for HCC are the need of the hour. KD is a therapy in which the dietary composition is altered, and is safer than drug therapy. Based on the available evidence, we speculate that KD may inhibit the growth of HCC by lowering blood glucose and insulin levels, regulating lipid metabolism, and alleviating the inflammatory response. Furthermore, KD can be considered as an adjuvant therapy for HCC. It has been found that KD intervention is effective in the early stages of the disease, but most patients are already in the middle to late stages when HCC is detected. Therefore, the use of KD prophylactically in the treatment of HCC is an issue that warrants further exploration. In addition to the timing of the intervention, the patient’s low compliance with KD is a major issue that should be addressed. Therefore, in future studies, the formulation of KD must be optimized to bridge the difference from the traditional diet, as well as to reduce the occurrence of adverse reactions. It is also necessary to increase patient education and support to improve compliance. Most importantly, there are few studies on the use of KD in HCC, and these do not fully reflect the therapeutic effects. Therefore, the long-term effects and safety of KD are remain unexplored. More studies with higher reliability should be designed to elucidate the mechanism of KD in the treatment of HCC and its application in clinical practice.

## Author Contributions

Conceptualization, YL. Original draft preparation, YL, CJ, XY, and CL. Review and editing, YL, CJ, and PK. Supervision, JS. Funding acquisition, JS. All authors have read and agreed to the published version of the manuscript.

## Funding

This research was funded by Chinese National Natural Science Foundation (Grant No. 81670567), and Scientific Research Fund of Zhejiang University (Grant No. XY2021030). Chaonan Jin was funded by China Scholarship Council.

## Conflict of Interest

The authors declare that the research was conducted in the absence of any commercial or financial relationships that could be construed as a potential conflict of interest.

## Publisher’s Note

All claims expressed in this article are solely those of the authors and do not necessarily represent those of their affiliated organizations, or those of the publisher, the editors and the reviewers. Any product that may be evaluated in this article, or claim that may be made by its manufacturer, is not guaranteed or endorsed by the publisher.
